# Sucrose stabilization of Respiratory Syncytial Virus (RSV) during nebulization and experimental infection

**DOI:** 10.1186/1756-0500-7-158

**Published:** 2014-03-18

**Authors:** Drew D Grosz, Albert van Geelen, Jack M Gallup, Shannon J Hostetter, Rachel J Derscheid, Mark R Ackermann

**Affiliations:** 1 Department of Veterinary Pathology, College of Veterinary Medicine, Iowa State University, 1600 S. 16th Street, Ames, IA 50011-1250, USA

**Keywords:** Respiratory Syncytial Virus, RSV, Nebulization, Sucrose

## Abstract

**Background:**

Respiratory syncytial virus (RSV) is a common respiratory pathogen that can cause severe pneumonia. *In vivo* studies of RSV can be difficult due to variation in viral infection and disease severity in some animal models. Factors that may contribute to the variation are decreases in viral titer due to preparation and storage and method of virus administration. Nebulization is one method of RSV administration that provides even distribution of virus to all lung lobes; however, the exact quantity of the virus killed by nebulization is not defined. To test the hypothesis that sucrose enhances RSV stability and infectivity, a series of *in vitro* experiments were conducted with RSV strain Memphis 37 stored at varying concentrations (0%, 3%, 5%, 8%, 10%, 15%, and 20%) of sucrose as a possible cryo- and nebulization protectant. The optimal *in vitro* concentration was then assessed *in vivo* in a lamb model.

**Methods:**

Prior to titering the virus on HEp-2 cells, the various virus solutions were subjected to one freeze-thaw cycle and one nebulization cycle. Forty-eight hours after viral plating, infectious foci were detected and counted using immunofluorescent imaging. Titers were determined after freeze-thaw and after freeze-thaw followed by nebulization, then compared to the stock titers (before freezing) as well as to one another to determine the loss of infectivity. To further test this *in vivo*, lambs 2 to 3-days-old were infected via nebulization with RSV using inoculate containing either 20% sucrose or no sucrose followed by assessments of infection severity.

**Results:**

Nebulization of virus in 0% sucrose resulted in a 0.580 log reduction in infectivity while virus in 20% sucrose exhibited a 0.297 log reduction. *In vivo* studies demonstrated that 20% sucrose enhanced RSV lesions and antigen distribution.

**Conclusions:**

The data suggests that both nebulization and freeze-thawing of RSV in the absence of sucrose cause unacceptable losses in viral infectivity and that sucrose acts as a RSV protectant in both regards.

## Background

Globally, respiratory syncytial virus (RSV) is estimated to cause 64 million cases of respiratory disease annually with the majority of cases occurring in children under the age of 5 and approximately 160,000 cases resulting in death [1-7]. In the United States, RSV is associated with an estimated 20-25% of pneumonia cases in children and 70% of bronchiolitis cases requiring hospitalization [1,2]. While much has been learned about the virus and disease pathogenesis, there are no fully effective vaccines or therapeutic regimens [8,9]. Animal models and *in vitro* studies have been instrumental in advancing knowledge of RSV pathogenesis; however, comparison of methodologies of studies is lacking.

Nebulization has been used as a method of RSV inoculation in various experimental animal models [10,11]. Other routes of inoculation include intranasal, intraocular, and intratracheal administration, while fiber optic bronchoscopic deposition has also been used successfully as a means of viral inoculation to specific regions of the lung (11). While each method has its advantages and disadvantages, nebulization results in a uniform distribution of virus throughout all lung lobes; however the extent to which nebulization damages or disrupts RSV has not yet been explored.

Sucrose has been used to protect RSV and other viral samples during shipping, storage and with swabs of virus [12-14]. The goals of this study were to: 1) determine the effects of freeze-thawing and nebulization on RSV stability/infectivity using RSV inoculum containing different concentrations of sucrose *in vitro* cultured in HEp-2 cells and, 2) test the extent to which the addition of sucrose to nebulized RSV inoculum affects RSV disease severity *in vivo* in perinatal lambs infected with a human strain of RSV. Lambs provide a good model of neonatal RSV infection. Ovine lung has similar development, structure, and cellularity to those of human infants [11]. Also, lambs are susceptible to human strains of RSV and develop lesions similar to those in human infants [11,15-17].

## Methods

### Experimental design of *in vitro* studies

#### Growing HEp-2 cells

HEp-2 cells were grown on 75 cm^2^ and/or 300 cm^2^ tissue culture flasks with a vent screw cap (TPP®) using 12 mL or 45 mL media, respectively, of Dulbecco’s Modification of Eagle’s Medium (DMEM) (Mediatech, Inc. Cat. No. 10-013-CV), 10% fetal bovine serum (FBS) (Atlanta Biologicals Cat. No. S11550), 50 μg/mL of kanamycin sulfate (Invitrogen Cat. No. 11815–032), and incubated at 37°C at 5% CO_2_ and 93% humidity. Cells were passaged 1:6 every 2–3 days, depending on cell confluence. Twelve-well cell culture plates (Costar®) were seeded with HEp-2 cells at a concentration of 2-3×10^5^ per well containing 6.5 mL of media. Cell concentration was determined prior to passaging by hemocytometer (Bright-Line®) to determine approximate cells per mL. Cells were allowed to grow overnight prior to infectious focus assay.

#### RSV M37 stock and sucrose

RSV Memphis strain 37 (RSV M37) was frozen at varying sucrose concentrations (0%, 3%, 5%, 8%, 10%, 15%, and 20%) to assess infectivity after cryo-storage and nebulization. HEp-2 cells were grown, as stated above, and infected with RSV M37. After 48 hrs, the virus was isolated from the cells after a freeze-thaw cycle (at −80°C) followed by centrifugation (2500 rpm for 10 min). 5 mL of the supernatant was then added to varying amounts of PBS (Sigma Cat. No. P3813-10PAK; 0.138 M NaCl, 0.0027 M KCl, pH 7.4 @ 25°C), 0% sucrose and 10% FBS solution or PBS, 60% sucrose and 10% FBS solution to attain desired sucrose concentrations of 0%, 3%, 5%, 8%, 10%, 15%, and 20%. The solution of 60% (w/v) sucrose in PBS was made, supplemented with 10% FBS and filtered using a Nalgene filter flask and used to accomplish the various desired final tested concentrations of sucrose mentioned above. A 0.5 mL aliquot was taken from each sucrose concentration and stored in a separate tube; this was used to determine the stock and post freeze-thaw titers of the virus. After preparation, samples were frozen at −80°C for about 3 weeks before use. Upon use, samples were thawed in a 37°C water bath just until ice disappeared.

#### Infecting HEp-2 cells with RSV M37 for infectious focus assay

Viral titers were assessed prior to freezing, after freezing, and following nebulization in a standard infectious focus assay. Collected condensates of nebulized vapors containing RSV M37 were used to infect HEp-2 cells. Vapors were collected using a nebulization apparatus coupled to a modified cold trap. The nebulization apparatus included: a nebulizer compressor unit, PARI nebulizer tubing, PARI Sprint™ nebulizer (PARI LC™), T-port connector, rubber stopper #3 (22 mm inside diameter), modified garden hose rubber washer (30 mm outer diameter and 22 mm inside diameter), and a 250 mL flask (PYREX®). 6 mL of RSV M37 inoculum containing the varying sucrose concentrations stated above, were individually loaded into the PARI Sprint^TM^ nebulizer and nebulized in three 2-mL amounts over a 23- minute period. A new nebulizer was used for each sucrose concentration. The condensed nebulized vapors within the 250 mL flasks were then diluted in duplicate with media in serial dilution fashion from 10^−1^ to 10^−5^. HEp-2 cells at a confluency of 75-85% (minimum of 50%) were used for the infectious focus assay. Prior to viral inoculation, the media within pre-seeded 12-well plates was discarded. 200 μL of diluted condensed viral vapor was then transferred to designated HEp-2 cell wells and allowed to incubate for 1 hr at 37°C to allow virus-to-cell contact. After 1 hr, 1 mL of media was added to each well and the plates were again incubated at 37°C for 48 hrs to allow for viral replication. Prior to freezing and post freezing, stock samples were also titered without the nebulization process; samples which came from aliquots of the original virus/sucrose preparations. Titers of these original pre-frozen viral stocks (with and without sucrose) ranged from 1.63 × 10^6^ to 2.91 × 10^6^ FFU/mL. Multiplicity of infection (MOI) for each study was generally between 0.5 and 1. All nebulization and plating was performed in a class II biosafety cabinet (NUAIRE®).

#### Determining RSV M37 infected cells

Immunofluorescence microscopy was used to determine cell infectivity. After the 48-hr incubation period, media within the wells was discarded and wells were rinsed once with TBStw [TBS-0.05% Tween 20, pH 7.4 (TBStw)]. Cells were then fixed with 0.5 mL 60% acetone/40% methanol solution for 1 min. The fixative was then removed and plates were allowed to dry for 2 min. Cells were rehydrated with 0.5 mL per well with TBStw and placed on a rotator (Boekel Scientific, model 260300 F) for 1 min after which well contents were discarded. A 1 mL blocking solution containing 3% bovine serum albumin (BSA) (Fisher Scientific, Hanover, IL) in TBStw was added to each well and plates were placed on the rotator for a minimum of 30 min, after which well contents were discarded. 325 μL of primary antibody [Meridian, MAb to RSV Fusion Protein, Cat. No. C87610M, Clone: RSV 3216 (B016)] containing 3% BSA-TBStw at 1:800 dilution was added to each well and plates were incubated overnight at 4°C on a rocker apparatus, after which well contents were discarded and wells were rinsed with TBStw. 325 μL of the secondary antibody (Invitrogen, Goat anti-Mouse Fab’ conjugated to AlexaFluor 488, Cat. No. A11017) containing 3% BSA-TBStw at 1:800 dilution was then added to each well and plates were placed on the rotator for a minimum of 30 min, after which well contents were discarded. Wells were then rinsed twice with 1 mL TBStw. 1 mL TBStw was then added to each well and fluorescing foci were visualized using an Olympus CKX41 inverted microscope with an external florescence bulb; X-Cite series 120 Q (Lumen Dynamics®). Cell aggregates ≥5 cells were counted as one infectious focus. Infectious foci per well were then converted to focus forming units (FFU) per mL using the following formula: [(arithmetic mean × dilution × 1000 μL/mL) / 200 μL assessed = FFU/mL]. Images were taken using an Olympus DP20 camera-microscope attachment.

### Experimental design of in vivo studies

Three groups of 2 to 3-day-old lambs (either sex; Polypay, Suffolk, Dorsett cross breed) were used for the study. RSV strain (Memphis 37) was grown in HEp-2 cells and delivered (1.25 × 10^6^ FFU/mL) to lambs in 6 mL culture medium using a PARI Sprint™ nebulizer (fitted with a cone-shaped face mask fitted with a rubber diaphragm that provided a seal around the mandible and nose) in three 2-mL increments over a 23 minute period. One group (n = 6 lambs) received this amount of RSV in culture media containing 20% sucrose whereas another group (n = 6 lambs) received this same amount of RSV in media lacking sucrose; control lambs (n = 2) received 6 mL culture medium (lacking virus) with 20% sucrose, also by nebulization. After inoculation with RSV, lambs in all groups received a daily antibiotic (Ceftiofur, 1–2 mg/kg body weight, intramuscular) to prevent secondary bacterial infections. Lamb temperatures, body weights, respiratory and heart rates were measured daily. Clinical severity of RSV disease for each lamb was assessed and scored as previously described [11,17,18]. Lambs were euthanized by sodium pentobarbital overdose on day 6 post-inoculation (p.i.) when RSV-induced lesions are maximal in this model [11,17,18]. Throughout the experiment, lambs received a custom diet (Milk Products Inc., Chilton, WI, USA) that lacked supplemental iodide, and the lack of iodide was verified using anion-exchange chromatography. The purpose of eliminating dietary iodide was to avoid any potential production of hypoiodite (OI^−^) which is a hypohalide generated by the ovine oxidative mucosal Duox/LPO/halide defense system in the presence of sufficient iodide. Hypoiodite has been demonstrated to be destructive to RSV [19]. All animal experiments were approved by the Institutional Animal Care and Use Committee (IACUC) of Iowa State University.

#### Post-mortem analysis of RSV disease severity

After euthanasia, the thorax was opened, lungs were removed, gross lesions scored as described previously [11,17,18] and photographed *in situ* and *ex vivo*. Tissue samples were collected from all lung lobes of each animal in a standardized manner. Multiple samples from each lobe were placed into cryovials and snap-frozen in liquid nitrogen for subsequent use in RNA isolation for hydrolysis probe-based reverse transcription real-time quantitative polymerase chain reaction (RT-qPCR). Two samples from each lobe were placed into tissue cassettes and fixed in 10% neutral-buffered formalin (NBF) for histological and immunohistochemical analysis, and two lung samples from each animal were placed into cryomolds and covered with CRYO-OCT Compound (Tissue-Tek, Torrance, CA) then stored at −80°C until cryosectioning. Immediately after lung removal, percentage parenchymal involvement was estimated for each lobe. Group averages were calculated to obtain the gross lesion score for each lobe [17,18].

In addition to the lung samples collected for RNA isolation and histological samples, bronchoalveolar lavage fluid (BALF) was collected from each lamb for determination of viral titers and cytology. BALF for viral titer assessment was obtained by flushing the right caudal lung lobe through a major bronchus with 5 mL of ice-cold modified Iscove’s media (42.5% Iscove’s modified Dulbecco’s medium, 7.5% glycerol, 1% heat-inactivated FBS, 49% DMEM, and 5 μg/mL kanamycin sulfate). 1 mL of each recovered BALF sample was then used immediately (never being frozen) for infectious focus assay. BALF for cytology (~0.5 mL from each lamb) was obtained by flushing a major bronchus of the accessory lobe with 1 mL PBS, pH 7.4. These BALF samples were submitted to the clinical pathology laboratory (Iowa State University, College of Veterinary Medicine) for total nucleated cell counts and slide preparation for cytology. An ADVIA120™ automated hematology analyzer was used to perform cell counts. Cytospin preparations of BALF were performed using a Shandon Cytospin 3 set at 800 rpm (samples were spun for 10 minutes with low acceleration). Slides were stained with modified Wright’s using a Hematek automated staining system. Differential cell counts (based on a 300 cell differential) were performed by a board-certified veterinary clinical pathologist blind to the identity of the samples.

Histologic scores were determined by evaluating percent consolidation and converting the observed percentage ranges to a simple integer based on a consolidation scale used by our laboratory previously (35, 36): 0% consolidation = 0, 1-9% consolidation = 1, 10-39% consolidation = 2, 40-69% consolidation = 3, 70-100% consolidation = 4 [17,18]. Group averages were calculated to obtain the alveolar consolidation score.

#### RNA isolation and RT-qPCR

Tissues from right and left cranial, right and left middle, and right and left caudal lung lobes (0.3-0.4 g of each) were homogenized in TRIzol (Invitrogen), pooled in equal (w/v) portions to create representative slurries for each animal, each of which was adjusted to 0.091 g/mL with additional TRIzol. Total RNA isolation continued as per manufacturer’s guidelines (Invitrogen) followed by DNase treatment (Ambion, TURBO DNase, Austin, TX) and diluting the isolates 1:10 with a combination of RNaseOUT (Invitrogen) and nuclease-free water (Ambion/Invitrogen). A NanoDrop (Thermo Fisher Scientific) was used to assess each sample for general RNA purity and quantity (A_260nm_/A_280nm_ all >1.98). In addition, Agilent Bioanalyzer 2100 analysis of the RNA routinely showed RIN values >8.0. RT-qPCR was carried out using One-Step Fast qRT-PCR Kit mastermix (Quanta, BioScience, Gaithersburg, MD) in a GeneAmp 5700 Sequence Detection System (Applied Biosystems, Carlsbad, CA) employing PREXCEL-Q for all set-up calculations [20-23]. Primers and probe for the RSV M37 nucleoprotein mRNA sequence targeted by the RT-qPCR in this study were designed using ABI Primer Express 2.0 based on RSV accession number M74568. Forward primer: 5’-GCTCTTAGCAAAGTCAAGTTGAACGA; reverse primer: 5’-TGCTCCGTTGGATGGTGTATT; hydrolysis probe: 5’-6FAM-ACACTCAACAAAGATCAACTTCTGTCATCCAGC-TAMRA. Prior to RT-qPCR, each 1:10-diluted total RNA sample was further diluted so that each final reaction contained 0.784 ng total RNA/μL; a dilution and sample concentration determined to be optimal by PREXCEL-Q [19,20]. The GeneAmp 5700 Sequence Detection System thermocycling conditions were 5 minutes at 50°C; 30 seconds at 95°C; and 45 cycles of: 3 seconds at 95°C and 30 seconds at 60°C. Samples and standards were assessed in duplicate wells and each quantification cycle (Cq) was converted to a relative quantity (X_0_r) based on a relative dilution standard curve using the equation: Xor = E_AMP_^(b ‒ Cq)^, where E_AMP_ and b are the exponential PCR amplification efficiency and the y-intercept values, respectively, obtained from a sample mixture-derived standard curve for RSV M37 nucleoprotein. The efficiency-corrected delta Cq (E_AMP_^ΔCq^) method was employed for final quantification calculations. Results were normalized to total tissue RNA loaded per reaction; identical for all reactions as per the PREXCEL-Q protocol. No-RT control reactions all proved negative for RSV and the assays were determined to be free of inhibition based on the PREXCEL-Q method [20-23].

#### Immunohistochemistry for RSV

Immunohistochemistry for localization, and relative quantification of RSV antigen was performed on formalin-fixed paraffin-embedded (FFPE) tissues [11,17,18]. After heating for 15 minutes at 58°C and standard deparaffinization through xylene and graded alcohols, antigen retrieval was performed using TE-0.05% Tween 20, pH 9.0 and a Decloaking Chamber™ Plus (Biocare Medical, Concord, CA). A temperature of 125°C was reached in about 18 minutes, after which the system cooled to 80°C after another 22 minutes. Blocking for 15 minutes with 3% bovine serum albumin (BSA) (Fisher Scientific, Hanover, IL) in TBS-0.05% Tween 20, pH 7.4 (TBStw) and blocking with 20% normal swine serum (NSS) (Gibco/Invitrogen) in TBStw for 15 minutes was followed by addition of a primary polyclonal goat anti-RSV (all antigens) antibody (EMD/Millipore/Chemicon, Billerica, MA) which was applied for 1.5 hours at room temperature (~22°C) at a dilution of 1:500 (8–10 μg/mL) in TBStw containing 10% NSS and 3% BSA. After TBStw rinses, biotinylated rabbit anti-goat secondary antibody (Kirkegaard-Perry Labs, Gaithersburg, MD) diluted 1:300 in TBStw containing 10% NSS and 3% BSA was applied for 45 minutes. Slides were rinsed, blocked for endogenous peroxidase activity (using 3% peroxide in TBStw) for 25 minutes, and streptavidin-conjugated HRP (Invitrogen) diluted 1:200 in TBStw was applied for 30 minutes. After rinsing slides with TBStw, Nova Red (Vector, Burlingame, CA) was applied for about 90 seconds followed by water rinses, counterstaining in Harris’ hematoxylin for 2 minutes, bluing with alkaline Scott’s water for 1 minute, standard dehydration through graded alcohols and xylene, and cover-slipping using Permount (Sigma, St. Louis, MO). Slides (containing two pieces of lung tissue each) were scored using the following procedure: 20 unique 10X fields on each slide were assessed for antigen staining and immunoreactive cells were counted within bronchioles and alveoli. The number of cells immunoreactive for RSV per field was then scored as percent lobular involvement based on the method used in our previous studies [17,18] without using the non-parametric 1–4 scale assignment.

#### Statistical analysis

All analyses were performed using GraphPad Prism 6 (GraphPad Software Inc, La Jolla, CA). RT-qPCR, IHC and gross lesion data was assessed by one-way ANOVA followed by Tukey’s post-test. All clinical data were assessed by two-way ANOVA, and cumulative weight change was additionally assessed by one-way ANOVA followed by Tukey’s post-test. Scored parameters were assessed using non-parametric (Kruskal-Wallis) and/or unpaired, two-tailed, non-parametric Mann–Whitney test analyses. Significance was accepted at *P* < 0.1 in these evaluations.

## Results

### *In-vitro* studies

**Figure 1:**
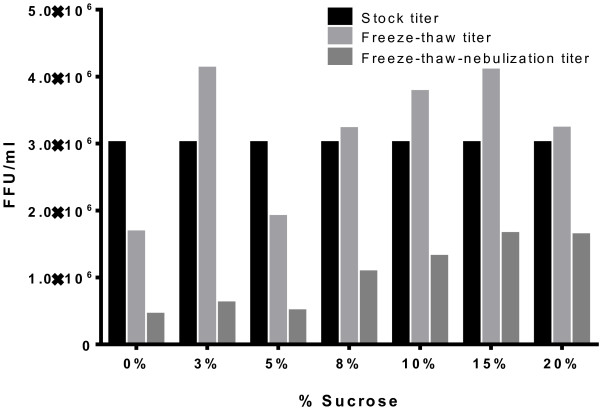
**Titer trends of M37 stock and M37 + sucrose solutions after freeze-thawing and freeze-thawing followed by nebulization.** All values have been factor-adjusted to a stock titer of 3 × 10^6^.

RSV in 0% sucrose concentration showed a 0.256 log drop (44.52% loss) in infectivity due to freeze-thawing and a 0.580 log drop (73.70% loss) due to nebulization; indicating an overall log drop of 0.836 (85.41% loss in infectivity) (Figure 1). RSV in 3% sucrose concentration showed no log drop (0.00% loss) due to freeze-thawing and a 0.831 log drop (85.25% loss) due to nebulization representing an overall log drop of 0.694 (79.75% loss) (Figure 1). RSV in 5% sucrose concentration showed a 0.198 log drop (36.61% loss) due to freeze-thawing and a 0.589 log drop (74.24% loss) due to nebulization yielding an overall log drop of 0.787 (83.67% loss) (Figure 1). RSV in 8% sucrose concentration showed no log drop (0.00% loss) due to freeze-thawing and a 0.477 log drop (66.67% loss) due to nebulization suggesting an overall log drop of 0.448 (64.38% loss) (Figure 1). RSV in 10% sucrose concentration showed no log drop (0.00% loss) due to freeze-thawing and a 0.461 log drop (65.39% loss) due to nebulization signifying an overall log drop of 0.362 (56.58% loss) (Figure 1). RSV in 15% sucrose concentration showed no log drop (0.00% loss) due to freeze-thawing and a 0.398 log drop (60.00% loss) due to nebulization indicating an overall log drop of 0.263 (45.46% loss) (Figure 1). RSV in 20% sucrose concentration showed no log drop (0.00% loss) due to freeze-thawing and a 0.297 log drop (49.55% loss) due to nebulization demonstrating an overall log drop of 0.266 (45.75% loss) (Figure 1).

### *In-vivo* studies

**Figure 2:**
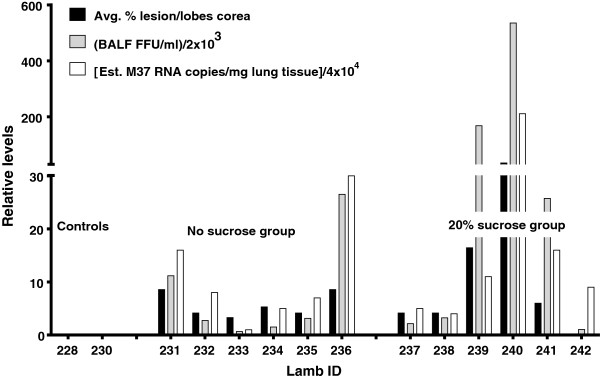
Gross lesion score, BAL titer of RSV and RNA levels of RSV detected in lung of lambs inoculated with RSV with and without sucrose.

**Figure 3:**
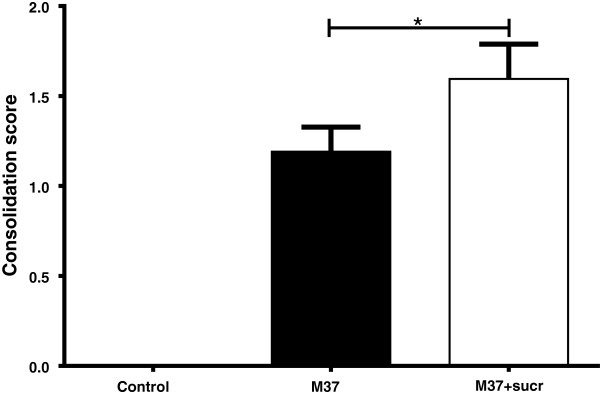
**Consolidation scores (histopathologic lesions) of lungs of lambs inoculated with RSV with and without sucrose by nebulization.** Lesions are significantly increased in lung of lambs inoculated with RSV containing sucrose compared to lambs receiving RSV lacking sucrose. Control lambs lacked lesions. Control n = 2; M37 n = 6; M37 + sucr n = 6. Error bars represent SEM, *P < 0.1.

**Figure 4:**
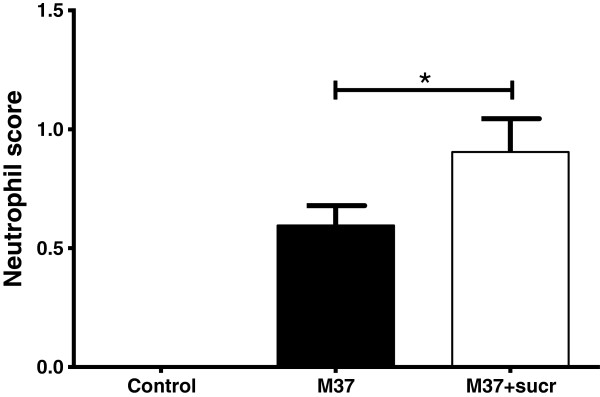
**Neutrophil infiltration scores into bronchioles of lungs of lambs inoculated with RSV with and without sucrose by nebulization.** Neutrophils levels are significantly increased in lung of lambs inoculated with RSV containing sucrose compared to lambs receiving RSV lacking sucrose. Control lambs lacked neutrophil infiltration. Control n = 2; M37 n = 6; M37 + sucr n = 6. Error bars represent SEM, *P < 0.1.

Clinically, there were no significant differences in expiratory effort, weight gain, temperature, heart or respiratory rates. There was a nearly-significant trend of increased gross lesion scores (11 ± 5.9) for lambs receiving RSV in 20% sucrose compared to lambs lacking sucrose (5.7 ± 0.95) (Figure 2) and consolidation scores of histopathologic lesions were significantly (*P* < 0.1) higher in lambs receiving RSV in 20% sucrose (1.6 ± 0.2 estimated number) compared to lambs receiving RSV lacking sucrose (1.2 ± 0.14 estimated number) (Figure 3). Also, histologically, neutrophil infiltration into infected bronchioles was significantly increased (*P* < 0.1) in lambs receiving RSV in 20% sucrose (0.9 ± 0.14 estimated number) compared to lambs receiving RSV without sucrose (0.6 ± 0.1 estimated number) (Figure 4). To evaluate the effect of RSV in 20% sucrose on inflammatory cell populations within the lung, differential cell counts were performed on BALF and significant differences in monocytes, lymphocytes, and neutrophils were not observed.

**Figure 5:**
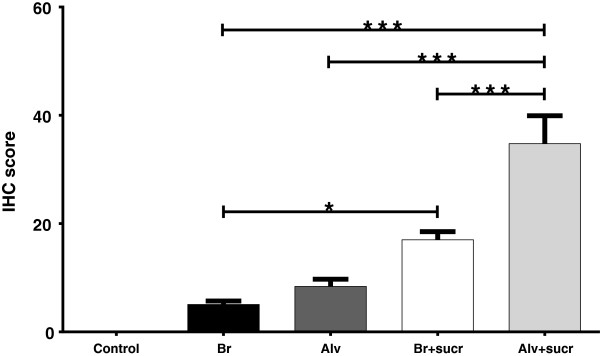
**RSV antigen levels in lungs of lambs inoculated with RSV with and without sucrose by nebulization.** RSV viral antigen levels are significantly increased in both bronchioles (Br) and alveolar (Alv) epithelium of lung of lambs inoculated with RSV containing 20% sucrose compared to lambs receiving RSV lacking sucrose. Control lambs lacked RSV antigen. Control n = 14; Br n = 42; Alv n = 42; Br + sucr n = 39; Alv + sucr n = 39. Error bars represent SEM, *P < 0.05, ***P < 0.001.

RSV viral titers from BALF fluid and RSV mRNA levels in lung trended higher in lambs receiving RSV in 20% sucrose compared to lambs receiving RSV lacking sucrose; controls lacked RSV viral titer and viral mRNA (Figure 2). RSV viral antigen detected by immunohistochemistry was significantly increased (*P* < 0.05) for antigen levels in bronchioles of lambs receiving RSV in 20% sucrose (17 ± 2.1) compared to antigen in bronchioles of lambs receiving RSV without sucrose (5 ± 0.9) (Figure 5). Also, RSV viral antigen scoring was significantly increased in alveolar epithelium (*P* < 0.001) in lungs of lambs receiving RSV in 20% sucrose (35 ± 7.3) compared to lambs receiving RSV without sucrose (8.4 ± 1.9) (Figure 5).

## Discussion

The use of sucrose to preserve and enhance infectivity/virulence and to transport respiratory syncytial virus (RSV) has been assessed previously but not, to our knowledge, with nebulization. We suspected that there could be a balance/give-and-take between the extent to which sucrose stabilization may protect RSV from damage by freeze-thaw, affect the virulence of the virus *in vivo* and *in vitro* and mitigate potential effects of the nebulization process itself. In 1968 it was shown that 44.5% sucrose was optimal for RSV storage at −70°C and that this concentration of sucrose in cotton swabs retained RSV viability for up to 7 days [12]. Another study has shown that 0.2 M sucrose phosphate resulted in significantly higher recovery of RSV as well as other viruses (cytomegalovirus, varicella-zoster virus, herpes simplex virus type 1) compared to 70% sorbitol [13]. Neither of these studies assessed nebulization nor did they assess animal infection with recovered virus.

In this study, the addition of sucrose to RSV M37 affected survival of virus during freeze-thawing, freeze-thawing followed by nebulization, and the extent of virulence *in vivo*. With freeze-thaw titers, there is an increase in RSV titer (virus survival) with the addition of sucrose concentrations compared to 0% (no) sucrose. The variability in stock and freeze-thawing titers resulted in some concentrations with freeze-thawing titers higher than stock; however, these were not statistically significant differences and we speculate that this might be caused by the breaking up of viral aggregates during the freeze-thaw process; thereby freeing up more infective virions prior to titering by infectious focus assay. The mechanism(s) by which sucrose may reduce viral loss with freeze-thaw could not be determined and was not further assessed in this study. For nebulization, a preservation of titer (e.g., increased RSV viability) occurred with higher sucrose concentrations compared to lower/no sucrose concentrations. Furthermore, even though RSV in 15 and 20% sucrose concentrations exhibited limited viral titer loss, some loss did occur at these concentrations. Given the nearly-significant trends in the titer data, sucrose seems to play a key role in RSV protection against the deleterious effects of both freeze-thawing and freeze-thawing followed by nebulization.

The delivery of RSV in sucrose to lamb lung by nebulization also had an effect on virulence *in vivo* as reflected by significantly increased consolidation scores (histopathologic lesions), neutrophil infiltration into bronchioles, and antigen levels (IHC scores). The reason(s) for the increased virulence is likely due, at least in part, to the increased survival of RSV throughout freeze-thawing and the freeze-thawing-nebulization process on account of the protective effect(s) of sucrose. Such increased preservation of viable virions would effectively increase the amount of infective virus deposited onto the respiratory mucosa. Although it is also possible that sucrose enhances attachment or maintains virus survival within the air-surface liquid (ASL) and/or positively affects some other aspects of RSV virulence, exploring such possibilities was not within the scope of this study. Sucrose may also affect droplet size and aggregation of viral particles which could alter the amount of virus per droplet and affect infectivity. Reasonable considerations for the positive effects on RSV stability include osmotic activity of sucrose in preserving the viral envelope, charge interactions between sucrose and the envelope, and bridging by sucrose of other protective media components to the viral envelope.

Although the addition of sucrose resulted in trends of increased gross lesions, RSV mRNA levels and BAL titers in infected lambs, these differences were not significant. This may be due to the timepoint post-infection (six days) at which lesions are maximal; however, it is possible that viral replication peaks at days 3–5 in lambs, as it does in certain other models [11,12,17,18]. Thus, it is possible that sucrose may have a significant effect on gross lesions, RNA levels and viral titers but perhaps earlier than the six-day timepoint post-nebulization. Because of the significance on some parameters and trends of enhanced infectivity in others, sucrose addition appears to be beneficial in preserving virulence in such studies.

## Conclusions

This study provides important information on methodology of *in vitro* and *in vivo* studies with a human strain of RSV and highlights the importance of carrying out proof-of-concept studies. This is especially true for studies in lambs which are out-bred animals and which are large enough to necessitate the use of larger volumes of virus. Optimizing virus stability and ensuring consistency in viral delivery are vital. While the nebulization method is effective in the lamb and other experimental animal models, these findings will also assist in comparison between studies in models that use different modes of inoculation.

## Abbreviations

RSV: Respiratory syncytial virus; RSV M37: Respiratory syncytial virus Memphis strain 37; DMEM: Dulbecco’s Modification of Eagle’s Medium; BALF: Bronchoalveolar lavage fluid; Sucr: Sucrose; FBS: Fetal bovine serum; BSA: Bovine serum albumin; TBS: TRIS-buffered saline; TBStw: TRIS-buffered saline + 0.05% tween 20; FFU: Focus forming units; IACUC: Institutional Animal Care and Use Committee; RT-qPCR: Reverse transcription quantitative polymerase chain reaction; NBF: Neutral-buffered formalin; Cq: Quantification cycle; X0r: Relative quantity; FFPE: Formalin-fixed paraffin-embedded; ANOVA: Analysis of variance; Br: Bronchiolar; Alv: Alveolar; IHC: Immunohistochemistry; SEM: Standard error of the mean.

## Competing interests

The authors declare that they have no competing interests.

## Authors’ contributions

DG completed the nebulization studies *in vitro*, AvG worked with DG on the *in vitro* studies and completed virology for both *in vitro* and *in vivo* studies, JG assisted with all studies and completed the IHC and RT-qPCR assays and graphing, SH completed the cytology assays, RD assisted with the design and animal studies and MA developed experimental designs, assisted with *in vitro* studies and much of the *in vivo* studies and completed the histopathology and immunohistochemistry assessments. All contributed to the assessment of the data and manuscript preparation. All authors read and approved the final manuscript.
